# Behind the Headache: A Case of Cerebral Venous Thrombosis Associated With Hormonal Contraceptive Use

**DOI:** 10.7759/cureus.87580

**Published:** 2025-07-09

**Authors:** Adélia F Sá, Bárbara G Moreira, Mariana S Alves, Rute C Gonçalves, Sandra Ferreira

**Affiliations:** 1 Family Medicine, Unidade de Saúde Familiar (USF) Gil Eanes, Unidade Local de Saúde (ULS) do Alto Minho, Viana do Castelo, PRT; 2 Obstetrics and Gynecology, Hospital de Santa Luzia, Unidade Local de Saúde (ULS) do Alto Minho, Viana do Castelo, PRT

**Keywords:** adverse effects, case report, cerebral venous thrombosis, combined hormonal contraceptives, contraceptive counseling, secondary headache, venous thromboembolism

## Abstract

Cerebral venous thrombosis is an uncommon but potentially life-threatening condition. We present the case of a previously healthy 26-year-old woman who developed cerebral venous thrombosis while using a combined hormonal contraceptive. She presented with progressive headaches unresponsive to analgesics. Imaging confirmed thrombosis in the left transverse and sigmoid sinuses and the left internal jugular vein. Anticoagulation was initiated with full resolution of symptoms and thrombus. Extensive thrombophilia screening was negative, and combined hormonal contraceptive use was identified as the main risk factor. The patient transitioned to a progestogen-only method. This case highlights the need to consider cerebral venous thrombosis in women with atypical headaches using combined hormonal contraceptives. It also emphasizes the importance of personalized contraceptive counseling based on thrombotic risk factors.

## Introduction

Venous thromboembolism (VTE) encompasses deep vein thrombosis (DVT), pulmonary embolism (PE), and cerebral venous thrombosis (CVT) [1,2]. CVT is a rare form of stroke, predominantly affecting young women [3-8]. It has a multifactorial etiology, with common risk factors including hormonal contraception, pregnancy, postpartum period, and heredity (e.g., factor V Leiden mutation) or acquired thrombophilias (e.g., antiphospholipid syndrome) [3,5-7]. Symptoms are often non-specific, with headache being the most prevalent clinical manifestation [3,6,7]. Diagnosis relies on neuroimaging, with anticoagulation as the first-line treatment. Prognosis is generally favorable [3-7,9].

The annual risk of VTE among women not using combined hormonal contraception (CHC) is approximately one to five per 10,000, compared to three to 15 per 10,000 among CHC users. This risk remains lower than that associated with pregnancy and the postpartum period [2,8,10-12]. This case report aims to highlight a CVT episode in a 26-year-old female patient and underscore the importance of appropriate contraceptive counseling in patients with special medical considerations and the necessity of a detailed clinical history in family planning consultations.

This article was previously presented as a poster at the 12th GO-MGF Meeting in Casa da Cultura de Paredes on November 28, 2024.

## Case presentation

We present the case of a 26-year-old Portuguese woman with no relevant personal or family medical history. She was previously healthy and did not smoke or consume alcohol. Her only regular medication was a combined hormonal contraceptive vaginal ring containing ethinylestradiol (0.015 mg/24 h) and etonogestrel (0.12 mg/24 h). On May 8, 2023, she presented to her family medicine practitioner with a change in the pattern of her chronic headaches, and a non-contrast cranial computed tomography (CT) scan was requested. The scan was performed on June 2, 2023, and showed heterogeneous and partially increased density of the left sigmoid sinus, along with mild peripheral densification of the ipsilateral internal jugular vein.

**Figure 1:**
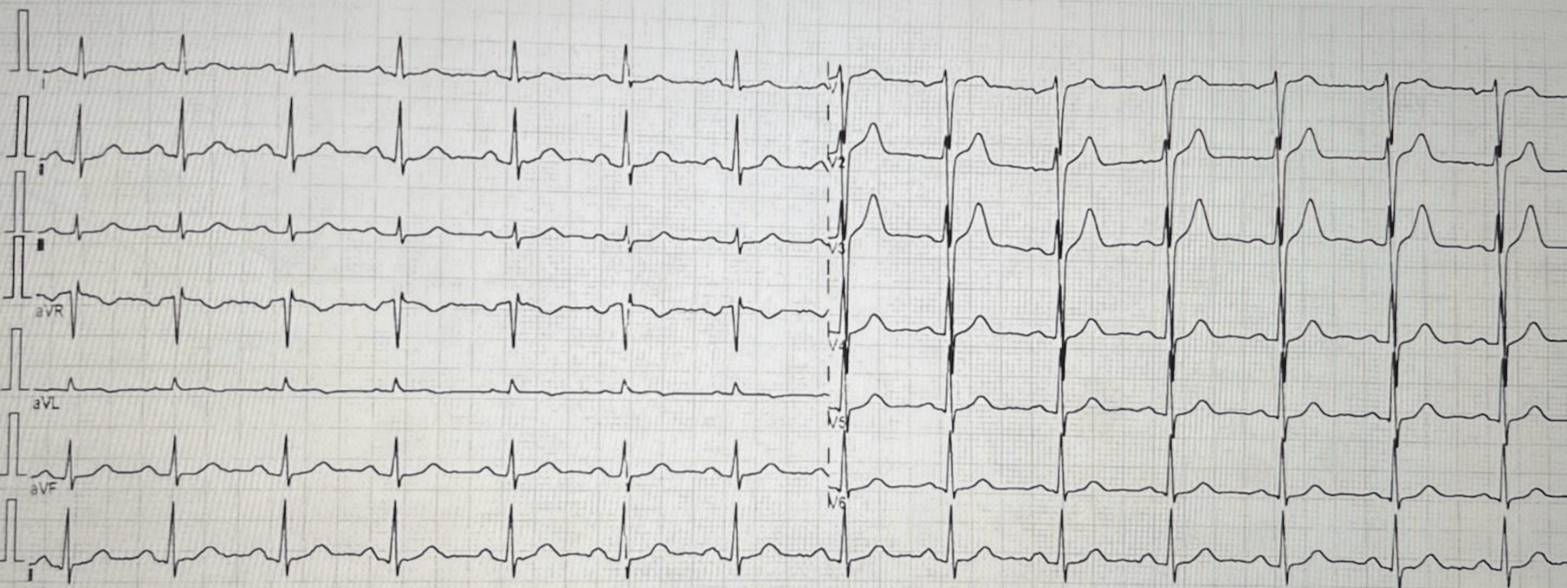
ECG performed in the emergency department. Twelve-lead electrocardiogram (ECG) demonstrating normal sinus rhythm at 84 bpm, with PR interval of 176 ms, QRS duration of 88 ms, and corrected QT interval (QTc) of 408 ms. No significant abnormalities observed.

On June 10, 2023, the reporting radiologist contacted the patient due to these imaging findings, which were suggestive of CVT, and referred her urgently to the emergency department. She was evaluated by internal medicine. The patient reported a two-month history of worsening headache, localized to the left frontal and ocular regions, described as pressing, with an intensity of eight out of 10 and refractory to standard analgesics (paracetamol and ibuprofen). Neurological and physical examinations were unremarkable. Initial investigations included comprehensive blood work (complete blood count, coagulation studies, and thrombophilia and autoimmune panels - Table 1), electrocardiogram (ECG) (Figure 1), and chest radiograph. None of these revealed any significant abnormalities.

**Table 1:** Laboratory results from June 10, 2023. MCV: mean corpuscular volume; MCH: mean corpuscular hemoglobin; MCHC: mean corpuscular hemoglobin concentration; RDW: red cell distribution width; gamma-GT: gamma-glutamyl transferase; AST: aspartate aminotransferase; ALT: alanine aminotransferase; HBsAg: hepatitis B surface antigen; anti-HBc: anti-hepatitis B core; anti-HBs: anti-hepatitis B surface; HCV: hepatitis C virus; TSH: thyroid-stimulating hormone; ANA: antinuclear antibody; ANCA: antineutrophil cytoplasmic antibody; INR: international normalized ratio; aPTT: activated partial thromboplastin time

Test	Result (2023-06-10)	Reference range
Red blood cells	4.03 × 10^12^/L	4.2-5.4
Hemoglobin	12.6 g/dL	11.8-15.8
Hematocrit	35.8%	36.0-46.0
MCV	88.8 fL	80.4-96.4
MCH	31.3 pg	26.7-30.7
MCHC	35.2 g/dL	31.7-35.7
RDW	12.4%	<15.0
Leukocytes	10.92 × 10^9^/L	4.0-10.0
Neutrophils	69.9%/7.6	55.0-75.0/1.5-8.0
Eosinophils	1.4%/0.2	1.0-3.0/0.0-0.3
Basophils	0.5%/0.1	0.0-2.0/0.0-0.3
Lymphocytes	21.6%/2.4	17.0-33.0/0.8-4.0
Monocytes	6.2%/0.7	5.0-9.0/0.0-1.2
Lymphocytes	21.6%/2.4	17.0-33.0/0.8-4.0
Monocytes	6.2%/0.7	5.0-9.0/0.0-1.2
Immature granulocytes	0.4%/0.0	0.0-3.0/0.0-0.3
Platelets	461 × 10^9^/L	150-400
Erythrocyte sedimentation rate	16 mm	4-10
Glucose	94 mg/dL	70-110
Urea	26.0 mg/dL	17.0-43.0
Creatinine	0.73 mg/dL	0.6-1.0
Sodium	140 mmol/L	136-145
Potassium	4.3 mmol/L	3.5-5.1
Alkaline phosphatase (ALP)	71 U/L	30-120
Gamma-GT	17 U/L	<38
AST	37 U/L	8-35
ALT	31 U/L	7-45
C-reactive protein	0.90 mg/dL	<0.51
HIV 1-2	Non-reactive	-
HBsAg	Non-reactive	-
Anti-HBc total	Non-reactive	-
Anti-HBs	Non-reactive	-
HCV antibodies	Non-reactive	-
TSH	0.97 µIU/mL	0.35-4.94
Total T4	12.09 µg/dL	4.87-11.72
IgA	154.0 mg/dL	60-400
IgG	973.0 mg/dL	700-1,600
IgM	102.0 mg/dL	40-230
ANA	1/160 (negative)	-
ANCA	1/20 (negative)	-
Anti-cardiolipin IgG	2 GPL-U/mL	Positive: >40, weakly positive: 10-40, negative: <10
Anti-cardiolipin IgM	10.0 MPL-U/mL	Positive: >40, weakly positive: 10-40, negative: <10
Anti-B2-glycoprotein I IgG	<0.6 U/mL	Positive: >10, clinically inconclusive: 7-10, negative: <7
Anti-B2-glycoprotein I IgM	<0.90 U/mL	Positive: >10, clinically inconclusive: 7-10, negative: <7
Prothrombin time (PT)	11.4 sec	11.0-13.2
INR	0.89	0.8-1.2
aPTT	30.4 sec	27.1-33.6
aPTT ratio	0.8	-
Fibrinogen	251 mg/dL	200-400
Lupus anticoagulant	1.2	0.8-1.2
Antithrombin (functional)	97.0%	83-128
Protein C (functional)	129%	70-150
Protein S (free Ag)	68.1%	55-124
Activated protein C resistance	2.88	2.6-3.3
Factor V Leiden mutation	Normal	-
Prothrombin gene mutation	Normal	-

**Figure 2:**
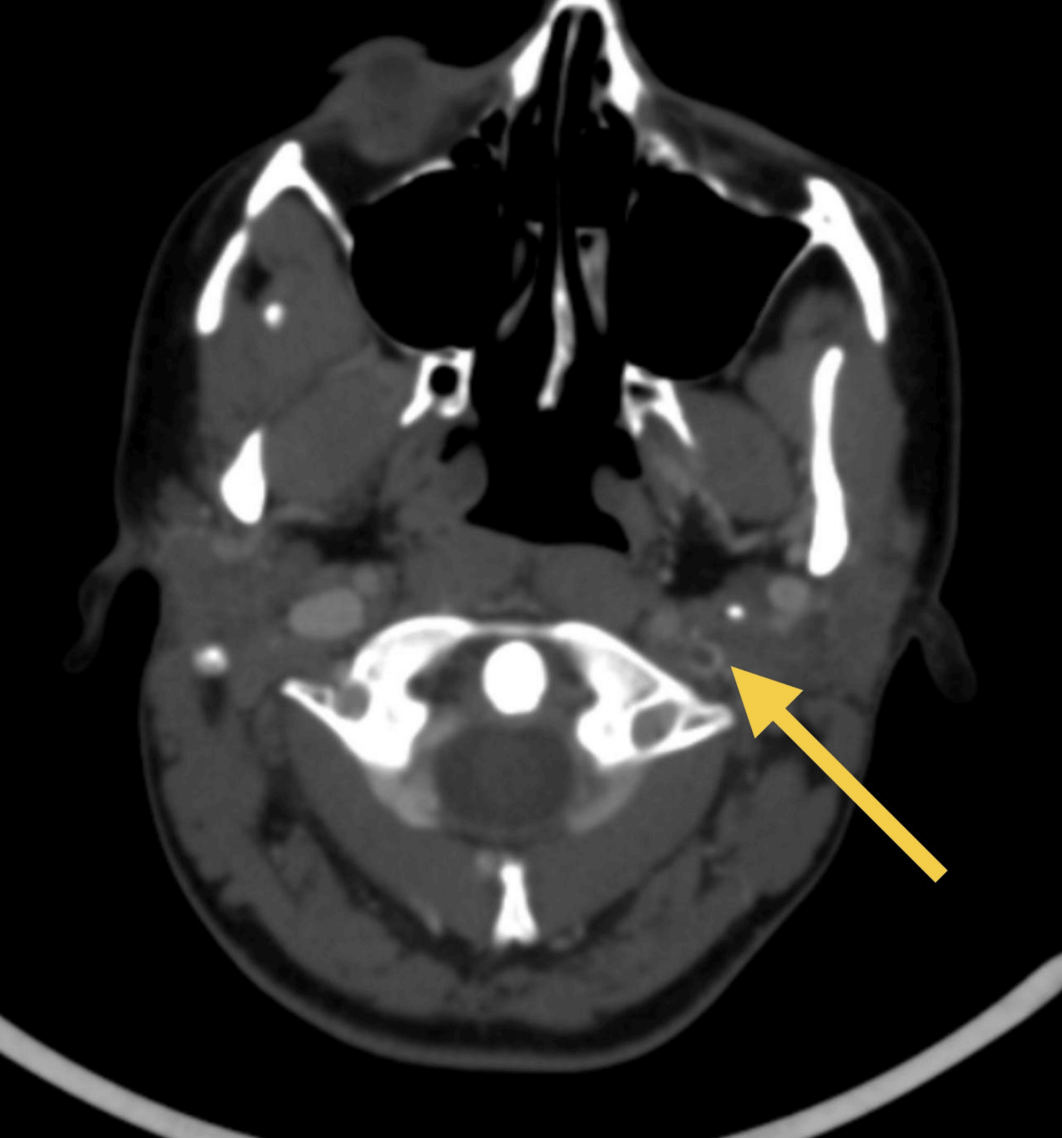
CT venography in the emergency department. Axial cerebral computed tomography (CT) venography showing a filling defect in the cranial segment of the left internal jugular vein (yellow arrow) consistent with venous thrombosis.

Cranial CT venography revealed thrombosis in the left transverse and sigmoid sinuses and the cranial segment of the left internal jugular vein (Figure 2).

Anticoagulation therapy with enoxaparin 50 mg every 12 hours was initiated, and the patient was hospitalized. The remaining blood test results, requested at admission, were reviewed during hospitalization and showed no abnormalities. She was subsequently switched to apixaban and discharged with follow-up appointments in internal medicine and gynecology. At discharge, apixaban 5 mg twice daily was maintained, and the CHC was discontinued.

During her gynecology consultation, the patient reported current use of condoms and prior use of combined oral contraceptives (ethinylestradiol 0.02 mg + gestodene 0.075 mg) for nine years, prior to switching to the vaginal ring. After counseling on contraceptive options suitable for patients with a history of thromboembolism, the patient opted for progestogen-only pills (desogestrel 0.075 mg).

In December 2023, repeat imaging showed complete resolution of the thrombosis. Anticoagulation was continued until March 2024. A repeated thrombophilia panel was again unremarkable (Table 2).

**Table 2:** Laboratory results from April 17, 2024. MCV: mean corpuscular volume; MCH: mean corpuscular hemoglobin; MCHC: mean corpuscular hemoglobin concentration; RDW: red cell distribution width; INR: international normalized ratio; aPTT: activated partial thromboplastin time

Test	Result (2024-04-17)	Reference range
Red blood cells	4.70 × 10^12^/L	4.2-5.4
Hemoglobin	14.6 g/dL	11.8-15.8
Hematocrit	40.7%	36.0-46.0
MCV	86.6 fL	80.4-96.4
MCH	31.1 pg	26.7-30.7
MCHC	35.9 g/dL	31.7-35.7
RDW	11.4%	<15.0
Leukocytes	6.43 × 10^9^/L	4.0-10.0
Neutrophils	64.4%/4.1	55.0-75.0/1.5-8.0
Eosinophils	0.3%/0.0	1.0-3.0/0.0-0.3
Basophils	0.3%/0.0	0.0-2.0/0.0-0.3
Lymphocytes	26.4%/1.7	17.0-33.0/0.8-4.0
Monocytes	8.4%/0.5	5.0-9.0/0.0-1.2
Immature granulocytes	0.2%/0.0	0.0-3.0/0.0-0.3
Platelets	367 × 10^9^/L	150-400
Anti-dsDNA	1/10 (negative)	-
Anti-cardiolipin IgG	2 GPL-U/mL	Positive: >40, weakly positive: 10-40, negative: <10
Anti-cardiolipin IgM	11.00 MPL-U/mL	Positive: >40, weakly positive: 10-40, negative: <10
Anti-B2-glycoprotein I IgG	<0.6 U/mL	Positive: >10, clinically inconclusive: 7-10, negative: <7
Anti-B2-glycoprotein I IgM	<0.90 U/mL	Positive: >10, clinically inconclusive: 7-10, negative: <7
Prothrombin time (PT)	11.9 sec	11.0-13.2
INR	1.03	0.8-1.2
aPTT	30.7 sec	27.7-36.4
aPTT ratio	0.9	-
Lupus anticoagulant	1.1	0.8-1.2
Antithrombin (functional)	110.0%	83-128
Protein C (functional)	121%	70-150
Protein S (free Ag)	79.7%	55-124
Activated protein C resistance	2.99	2.6-3.3

The thrombotic event was attributed to CHC use, and apixaban was discontinued. Four months later, follow-up laboratory work remained normal, and the patient was discharged from specialist care.

## Discussion

VTE is a serious but rare adverse effect of CHC [2,8,10,11,13,14]. Risk varies with estrogen type and dosage, with higher risk associated with ethinylestradiol-containing contraceptives [11,14]. The first year of use carries the greatest risk, which decreases with prolonged use [2,8,10,12]. Progestogen-only contraceptives are not associated with increased VTE risk [8,11,13-15]. Other contributing risk factors include age, body mass index, smoking, immobility, and inherited or acquired thrombophilias [1,2,8,10-12,16].

CVT is a rare cause of stroke that predominantly affects young women due to CHC use [1,3-8]. Clinical presentation varies but frequently includes subacute headache, papilledema, focal neurological deficits, seizures, altered mental status, or coma [3,5-7].

Initial imaging in the emergency setting often begins with non-contrast cranial CT, given its rapid availability. Although it may be normal in a significant proportion of cases, non-contrast CT can occasionally demonstrate indirect or direct signs of CVT, such as venous infarction, cerebral edema, hemorrhage, or spontaneous hyperdensity of thrombosed sinuses. Magnetic resonance imaging (MRI) with MR venography remains the preferred diagnostic modality, due to its superior sensitivity and ability to directly visualize venous flow. Alternatively, contrast-enhanced CT venography is a valid and widely used option, particularly when MRI is unavailable or contraindicated [3,5-7,9].

The first-line treatment is anticoagulation with low-molecular-weight heparin (LMWH), followed by oral anticoagulation using either vitamin K antagonists or direct oral anticoagulants (DOACs). The recommended treatment duration typically ranges from three to 12 months, depending on the underlying etiology [3-7,9]. In cases where the thrombotic event is associated with CHC, a shorter course of anticoagulation, typically three to six months, is considered reasonable, given the apparently low risk of recurrence in this context [17,18]. The prognosis is usually favorable, with the majority of patients achieving full recovery [3,5-7].

CHC (oral, transdermal, or vaginal) is contraindicated in women with a history of VTE [2,15,16,19]. Suitable alternatives include progestogen-only methods, copper intrauterine devices (IUDs), or non-hormonal options [2,8,13-16,19]. Injectable progestins are contraindicated in women with high VTE recurrence risk (e.g., idiopathic VTE, CHC-related VTE, pregnancy-associated VTE, recurrent VTE, thrombophilia, or malignancy) [2,13,15,16].

Anticoagulation increases the risk of abnormal uterine bleeding and hemorrhagic ovarian cysts. Therefore, copper IUDs are generally less favorable in this context. Progestogen-only contraception may offer added gynecological benefits, with the levonorgestrel IUD being particularly advantageous [2,16]. Superficial venous disorders should also be considered: CHC is contraindicated in patients with a history of superficial vein thrombosis but may be used in those with varicose veins [2,16].

This case demonstrates appropriate diagnosis, treatment, and contraceptive management in alignment with current guidelines. In this patient, CHC was the only identifiable VTE risk factor, indicating low recurrence risk. She remains asymptomatic without thrombotic sequelae.

## Conclusions

CVT, although rare, requires a high index of suspicion. This is particularly true in women presenting with atypical headaches and established risk factors for VTE, such as CHC use. Prompt recognition and timely anticoagulant therapy are essential for favorable clinical outcomes and minimizing long-term complications.

This case reinforces the importance of individualized contraceptive counseling based on a comprehensive medical and gynecological history. While CHCs remain a generally safe and effective option for most women, identifying those at increased thrombotic risk is crucial to guiding safer alternatives and optimizing reproductive healthcare.
